# Symmetric Allosteric Mechanism of Hexameric *Escherichia coli* Arginine Repressor Exploits Competition between L-Arginine Ligands and Resident Arginine Residues

**DOI:** 10.1371/journal.pcbi.1000801

**Published:** 2010-06-03

**Authors:** Rebecca Strawn, Milan Melichercik, Michael Green, Thomas Stockner, Jannette Carey, Rüdiger Ettrich

**Affiliations:** 1 Chemistry Department, Princeton University, Princeton, New Jersey, United States of America; 2 Department of Structure and Function of Proteins, Institute of Systems Biology and Ecology, Academy of Sciences of the Czech Republic, and Institute of Physical Biology, University of South Bohemia, Nove Hrady, Czech Republic; 3 Biology Department, The College of New Jersey, Ewing, New Jersey, United States of America; 4 Department of Medical Chemistry, Medical University of Vienna, Vienna, Austria; National Cancer Institute, United States of America and Tel Aviv University, Israel

## Abstract

An elegantly simple and probably ancient molecular mechanism of allostery is described for the *Escherichia coli* arginine repressor ArgR, the master feedback regulator of transcription in L-arginine metabolism. Molecular dynamics simulations with ArgRC, the hexameric domain that binds L-arginine with negative cooperativity, reveal that conserved arginine and aspartate residues in each ligand-binding pocket promote rotational oscillation of apoArgRC trimers by engagement and release of hydrogen-bonded salt bridges. Binding of exogenous L-arginine displaces resident arginine residues and arrests oscillation, shifting the equilibrium quaternary ensemble and promoting motions that maintain the configurational entropy of the system. A single L-arg ligand is necessary and sufficient to arrest oscillation, and enables formation of a cooperative hydrogen-bond network at the subunit interface. The results are used to construct a free-energy reaction coordinate that accounts for the negative cooperativity and distinctive thermodynamic signature of L-arginine binding detected by calorimetry. The symmetry of the hexamer is maintained as each ligand binds, despite the conceptual asymmetry of partially-liganded states. The results thus offer the first opportunity to describe in structural and thermodynamic terms the symmetric relaxed state predicted by the concerted allostery model of Monod, Wyman, and Changeux, revealing that this state is achieved by exploiting the dynamics of the assembly and the distributed nature of its cohesive free energy. The ArgR example reveals that symmetry can be maintained even when binding sites fill sequentially due to negative cooperativity, which was not anticipated by the Monod, Wyman, and Changeux model. The molecular mechanism identified here neither specifies nor requires a pathway for transmission of the allosteric signal through the protein, and it suggests the possibility that binding of free amino acids was an early innovation in the evolution of allostery.

## Author Summary

A controversial prediction of the famous allostery model of Monod, Wyman, and Changeux is that constraints imposed on protein subunits by multimerization are relaxed by ligand binding, but with conservation of symmetry in partially-liganded states. Interpretation of thermodynamic ligand-binding data through the lens of molecular dynamics simulation has led to structural and energetic description of such a state for the hexameric *Escherichia coli* arginine repressor, which displays strong negative cooperativity of L-arginine binding. The results indicate that partially-liganded states can be structurally symmetric despite their conceptual asymmetry. The symmetric relaxed state is visualized as a multimer with all subunits anchored near the center, and with motions transferred to the periphery of the assembly. Thus, even during sequential filling of binding sites, symmetry can be maintained by exploiting the dynamics of the assembly and the distributed nature of its cohesive free energy.

## Introduction

**Figure 1:**
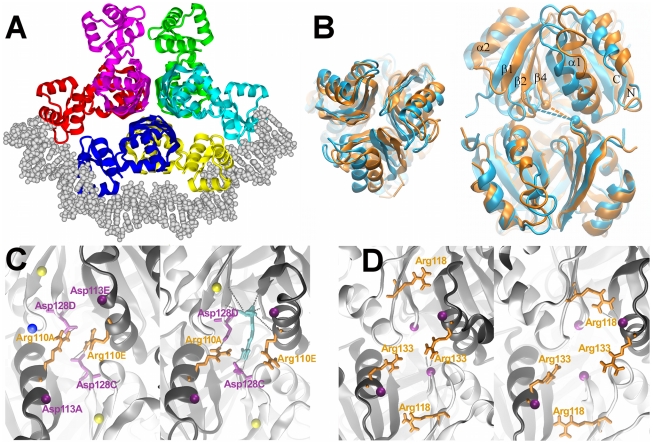
Structural features of ArgR. **A. Intact ArgR in complex with DNA.** The protein is viewed down the three-fold axis, with central ArgRC domains forming two trimers stacked on top of each other, and six peripheral ArgRN domains docked with bent B-form DNA. Subunit colors are yellow, A; red, B; green, C; blue, D; cyan, E; magenta, F. The protein structure was prepared from PDB ID 1B4A (apoBstArgR) and DNA structure as described in [13] from PDB ID 1J59 (cAMP receptor). **B. ArgRC rotation.** Overlay of average structures of the ArgRC domain hexamer from the equilibrated part of the simulations of holoArgRC (orange) and apoArgRC (blue). The overlay was prepared by C_α_ RMSD minimization using the bottom (ABC) trimer of each structure to show the conformational shift that occurs uniquely in apoArgRC, in which one trimer rotates clockwise relative to the other. Left, top view in approximately the same size and viewpoint as in panel A. Right, side view. Selected secondary structure elements and N- and C-termini are labeled for orientation. CPK spheres mark C_α_ atoms of the Gly103-Asp128 residues whose interatomic distances (dashed lines) are measured to quantify rotation. **C, D. L-arg binding sites.** The view is approximately that of panel B, right, but zoomed in on the trimer interface, showing four subunits in front with the two in back faded for clarity. The viewpoint is fixed in all panels by using C_α_ RMSD minimization to overlay the dark grey helix shown at lower left. Each panel shows one snapshot that resembles the mean state of each indicated simulation. **C. EcArgRC.** Left, apoArgRC. Right, apoArgRC +1arg. Arg110 forms a doubly-hydrogen bonded salt-bridge (dashed lines) with Asp128. Arg and Asp residues are identified by number and by subunit as defined in panel A. For clarity, filled circles indicate approximate locations of some residues, and some are unlabeled: His99 (yellow), Asp113 (purple), Gly103 (blue). L-arg ligand, cyan; dashed lines indicate some of the interactions made by the Cα substituents of L-arg, with others omitted for clarity. **D. ApoMtArgRC.** Two pairs of Arg-Asp salt bridges (dashed lines) permit rotation in both directions. Left, Arg133-Asp146 promotes clockwise rotation. Right, Arg118-Asp132 promotes counterclockwise rotation.

Arginine repressor (ArgR) is the master regulator of the arginine regulon in a wide variety of bacteria [1], acting as direct sensor and transcriptional transducer of intracellular L-arginine (L-arg) concentrations to provide feedback control over biosynthesis and catabolism of L-arg. The co-effector L-arg binds to a central hexamerization domain, altering DNA affinity and specificity [2] of peripheral domains (Figure 1A). Thus, although its activation mechanism is unknown despite decades of study, ArgR is an apparent example of the action-at-a-distance principle embodied in the concept of allostery [3]. The structural organization of ArgR into N- (ArgRN) and C-terminal (ArgRC) domains, and the functional division of labor between them, are conserved even among distant orthologs that display an unexpected diversity of reported biochemical properties, notably the L-arg dependence of hexamerization and DNA-binding equilibria [4]–[8]. An allosteric mechanism was inferred by comparison of crystallized, intact, unliganded apoprotein from the thermophile *Bacillus stearothermophilus* (apoBstArgR) with its liganded C-terminal domain fragment (holoBstArgRC), which differ by ∼15 degrees rotation about the trimer-trimer interface that was ascribed to L-arg binding and presumed to be transmitted to the DNA-binding domains [9]. However, rotation could reflect crystallization conditions and/or crystal packing, or the differential presence of the N-terminal domains, and no structure of apoBstArgRC has been reported. A similar degree of rotation was reported recently between apo- and holoArgRC of *Mycobacterium tuberculosis* (MtArgRC) [10], but the relationship between rotation and the molecular mechanism of L-arg allostery is still unclear. Pursuit of the allosteric mechanism of *E. coli* K-12 ArgR (EcArgR), the most thoroughly studied ArgR, is motivated by a wealth of genetic, biochemical, and biophysical knowledge [1], [2], [4], [11]–[16] that is unavailable for any ortholog and is expected to constrain activation models.

Unlike the *Bacillus* and *Mycobacterium* proteins, crystal structures of the *E. coli* ArgR C-terminal domain with (holoEcArgRC) and without (apoEcArgRC) bound L-arg are essentially identical [14], with maximum local shifts of <∼0.5 Å except at poorly-ordered monomer termini, and C_α_ RMSD ∼0.76 Å for apo- and holo- hexamers, a value similar to the RMSD for monomers within holoEcArgRC. Crystalline apo- and holoEcArgRC hexamers are also entirely symmetric, a finding that is seemingly incongruent with the complex thermodynamics of L-arg binding. Isothermal titration calorimetry (ITC) confirms that EcArgR and EcArgRC hexamers bind six equivalents of L-arg, but with a negatively cooperative binding mechanism in which the first binding event has ∼100-fold greater affinity than the subsequent five events [16]; thus L-arg binding is itself allosteric, even in the absence of DNA binding or DNA-binding domains. Negative cooperativity with such different affinities creates two action levels in the protein's response to L-arg, although it is not yet known how these levels are exploited by multifunctional ArgR in carrying out its physiological roles. For both ArgR and ArgRC, binding of the first L-arg, though exothermic overall, is associated quantitatively with a slowly-evolving endothermic heat flow, as is binding of the analog L-canavanine despite almost 1000-fold weaker affinity than L-arg. Such slow, offsetting heat flows have been suggested [16] to be a hallmark of global conformational response that may underlie the allosteric transition.

Further interpretation of this complex system requires an understanding of the protein's conformational landscape. Molecular dynamics (MD) simulations of the ∼50,000 Da ArgRC domain with and without bound L-arg provide a picture of populated conformations that identifies the molecular origins of its negative cooperativity and explains its ITC endotherm. The results reveal that, despite the conceptual asymmetry of partial ligation, the symmetric relaxed state predicted by the model of Monod, Wyman, and Changeux (MWC; [17]) can be achieved by exploiting the dynamic nature of the assembly and the distributed nature of its cohesive free energy. The allosteric mechanism discovered here neither specifies nor requires pathways or networks through the protein to transmit the allosteric signal among L-arg binding sites, and it represents a structurally and thermodynamically explicit and particularly simple example of the now-common view [18], [19] that the emergent property of allostery derives from inherent, universal dynamics of proteins and that allosteric effectors redistribute protein conformational ensembles.

## Materials and Methods

Molecular dynamics analysis used the modeling package GROMACS 3.3.1 [20], [21]. ArgRC apo- and holoprotein crystal structures (PDB entry 1XXC and 1XXA, respectively) were prepared by standard methods in YASARA [22] including the following steps, and solvated in SPC water [23]. The simulation cell extended 10 Å beyond the protein, and periodic boundary conditions were applied. The system was neutralized with 36 sodium ions. For simulations the GROMOS87 force field [24] was employed with corrections [25], [26]. Electrostatics were evaluated using the particle-mesh Ewald method [27] with a cutoff of 10 Å. van der Waals forces were evaluated with a Lennard-Jones potential having an 18 Å cutoff to permit L-arg ligands separated by 17 Å in adjacent binding sites to ‘feel’ each other; essentially identical results were obtained in trial calculations with the standard cutoff of 14 Å (data not shown). Weak temperature and pressure coupling [28] were employed (coupling constants 0.1 ps), with the protein and solvent atoms having separate baths maintained at 300 K, and pressure maintained at 1 bar with a compressibility of 4.6*10^−5^/bar. Virtual-site hydrogens [29] were employed to increase calculation speed by allowing for time steps of 5 fs. Bond lengths were constrained using LINCS [30]. The neighbor search list was updated every 20 fs. The solvated system was first energy minimized using steepest descent and the solvent was allowed to relax for 250 ps while keeping the protein restrained. Initial Boltzmann- weighted velocities were generated randomly and the system was further equilibrated for 500 ps. The MD production runs without constraints were carried out at least for 20ns and in some cases were continued for additional 50 ns.

DynDom [31], [32] was used to carry out a protein domain motion analysis that allows identification of moving domains, defines the screw axis, and measures the degree of rotation between two conformers. A sliding window length of 11 and an intra- to interdomain rotation ratio of 0.7 were used to compare the last frames of trajectories to the starting frames and to each other.

Principal-components analysis was used to identify the global motions of the ArgRC hexamer. The elements of the covariance matrix are defined as
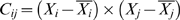
where i and j are atom indices, 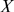 are the coordinates and 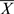 is the reference structure. The correlation of atomic displacements of Cα atoms was analyzed by calculating the atomic covariance matrix, defined as the sum (C_xx_ + C_yy_ +C_zz_) of atom pair covariances (C_ij_) in the X, Y and Z directions, respectively. The reference structure is represented by the mean values of the coordinates of each Cα atom over the analyzed trajectory. In the analyses of rotation from the crystal structure referenced to one trimer (made up monomers ABC), the initial structure of each simulation was used as reference instead of the average structure. Eigenvectors representing molecular motions are extracted from the covariance matrix by diagonalization; the eigenvectors with largest amplitude represent the motions that describe global conformational changes. These motions are visualized by extracting coordinates representing the extreme conformations along the eigenvectors.

Binding free energies were computed by the linear interaction energy method using *g_lie* [20], with an electrostatic term for ligand/water interaction of −453.458 kJ/mol and a Lennard Jones term for ligand/water interaction of −4.63 kJ/mol that were calculated from 500 ps simulations of L-arg in SPC water utilizing the same force field parameters as for the whole system. The default values of the scaling factors for the Lennard-Jones term (α = 0.18) and for the electrostatic term (β = 0.50) were used as they are valid for small, charged molecules like L-arg [33]–[35]. Ligand binding enthalpies were calculated as the nonbonded interaction energy by *g_energy* [20]. The interaction energy between the free ligand and water is subtracted from the sum of the interaction energy terms calculated by the force field between protein and bound ligand and bound ligand and water.

Root mean square deviations (RMSD) were calculated by *g_rms* [20] for the whole trajectory taking as a reference the coordinates at the start of the simulations. To calculate the atomic fluctuations (RMSF), the trajectory was separated into six independent trajectories, one for each monomer, and the root mean square fluctuation was then calculated by *g_rmsf* from the last 10 ns of the simulations. Covariance matrices were calculated with *g_covar* and processed by *g_anaeig* [20] that performs a principal-components analysis to determine the major movements. The last 2 ns of the trajectories were compared to the mean structure of the last 2 ns.

Entropies were computed from the mass-weighted covariance matrices produced by *g_covar* using a quasi-harmonic approximation [36] implemented in the *calc_entropies.pl* script from the Gromacs web-page (www.gromacs.org). Differences of the conformational entropy were calculated by quasi-harmonic analysis from k_B_/2(det σ_a_/det σ_b_), where detσ_a_ and detσ_b_ are covariance matrices of atomic fluctuations, and k_B_ is Boltzmann's constant. To gain higher accuracy the original Schlitter's approximation [37] is improved by removing the singularity of the covariance matrix in Cartesian coordinates [38]. Entropy calculations were carried out over the last 10 ns of the trajectories of three independent 20-ns simulations, permitting calculation of the standard deviation for each entropy value. Frames were sampled every 0.1 ps, well beyond the minimum frame number required for quasi-harmonic approximations [38], yielding results that are independent of frame number. Each structure in the trajectory (every frame) was aligned to the average hexamer structure by mass-weighted fitting of heavy atoms to remove translational and rotational degrees of freedom, but not trimer rotation. All atoms (2850) were included in the analysis with the exception of the virtual sites, which do not contribute to the degrees of freedom as their position is reconstructed at each MD integration step.

Radius of gyration was computed by *g_gyrate* [20]. The distances between selected atoms and between centers of mass were calculated by *g_dist* and graphs were prepared in Grace (http://plasma-gate.weizmann.ac.il/Grace/). The histograms of distances between selected atoms were calculated in Calc Spreadsheet included in the OpenOffice suite (http://openoffice.org). For structure and trajectory visualization Yasara [22] and VMD [39] were used.

## Results/Discussion

### Structures and simulations

Multiple alignment of 500 intact ArgRs (not shown) suggests that the extremely variable sequence preceding the tandem ββα repeats of the ArgRC fold, which forms an extra helix in the apoBstArgRC crystal structure [10] that has no equivalent in the *E. coli* ArgR sequence, belongs not to the ArgRC fold as suggested for BstArgR but to a highly variable interdomain linker. Thus, *E. coli* apo- and holoArgRC crystal structures (PDB IDs 1XXC and 1XXA, respectively [14]) containing residues 80–156 of intact ArgR are inferred to contain the entire C-terminal domain of EcArgR. These PDB files were prepared as initial structures for simulation as described in Materials and Methods. Independent replicates of these starting structures were derived by removing all six L-arg ligands from 1XXA (holoArgRC-6) and by adding six L-arg to 1XXC (apoArgRC+6) in order to probe the reaction coordinate from opposite directions. Intermediate ligation states were prepared by adding one L-arg in turn to each monomer of 1XXC (apoArgRC+1) to form six singly-liganded starting structures; six more were prepared by removing five L-arg from 1XXA in all permutations (holoArgRC-5); and fifteen doubly-liganded starting structures were prepared by adding two L-arg to apoArgRC in every permutation. Simulations using GROMACS ([20]; Materials and Methods) ran for a minimum of 20 ns using steps of 5 fs, facilitated by using virtual-site hydrogens [29]. A Lennard-Jones potential having an 18 Å cutoff was used to permit L-arg ligands, which are separated by 17 Å in adjacent binding sites, to ‘feel’ each other; essentially identical results were obtained in trial calculations with the standard cutoff of 14 Å (not shown), indicating that L-arg ligands do not experience direct pairwise interactions.

Root-mean-square deviations (RMSDs) relative to the starting structures (Figure S1) tend toward values of ∼2 Å, typical for equilibrated systems of this size [40]. Stable plateau values are reached by ∼10 ns except for holoArgRC-6 and apoArgRC+6 RMSDs that drift slightly through 70 ns. Monomer mass distributions and radii of gyration also equilibrate by ∼5 ns (not shown). C_α_ root-mean-square fluctuations (RMSFs) relative to the hexamer structure averaged over the last 10 ns display maximum values of ∼1.5 Å for loop residues and minimum values of ∼0.3 Å for secondary structure segments (not shown), corresponding well with the pattern of crystallographic B-factors in the PDB files.

### Arg residues promote rotation

Manual inspection of the apoArgRC trajectory reveals that a dramatic shift away from the starting structure occurs very early in the equilibration phase. Quantitative analysis of domain motions as described in Materials and Methods indicates that this early shift comprises clockwise rotation of one apoArgRC trimer with respect to the other by ∼13° (Figure 1B). Rotation in the counterclockwise direction does not occur; the clockwise direction of rotation is opposite of that observed in the *B. stearothermophilus* and *M. tuberculosis* crystal structures. Independently repeated runs using comparable preparation and equilibration steps with a wide range of force fields (Gromos96, GMX, OPLS-AA, Amber 96, Amber99, Yamber2), two different water models (SPC, TIP3), and different randomly-assigned initial velocities all result in the same shift (not shown). These findings, together with the fact that the shift was never observed in simulations with holoArgRC, argue strongly against artifactual causes of trimer rotation.

ApoArgRC simulations equilibrate after the early conformational shift but the starting conformation is not visited again, suggesting that crystals trap a high-energy species that is rare in solution. Yet only minor differences are detected upon structural comparison of the rotated conformation with the starting conformation. Rotation alters the apposition of apoArgRC monomers across the inter-trimer interface so that Leu82, Leu85, Ala104, and Leu107 face Leu107, Pro102, Ser101, and Leu85 instead of the four symmetry-equivalent residues. Both interfaces are uniformly planar and similarly hydrophobic, and no structural changes are propagated beyond the interface. Only minor differences in the nature and extent of solvent-exposed surface area accompany rotation.

Visual inspection of the trajectory indicates that the early rotational event is correlated with an altered conformation of the Arg110 sidechain in all six subunits (Figure 1C). In the rotated conformation each Arg110 sidechain extends almost completely into each ligand-binding pocket, facilitated by rotation about dihedral angle C-Cα-Cβ-Cγ from ∼23±59° (average±s.d. in apoArgRC crystal structure) to ∼170±14° in rotated apoArgRC, and the Arg110 guanidino group makes a bidentate, doubly hydrogen-bonded salt bridge with the Asp128 carboxylate lying diagonally across the trimer interface. This interaction is equivalent to the interaction made by each L-arg guanidino group in holoArgRC crystals and simulations, where no Arg110-Asp128 interactions are observed and Arg110 residues face the solvent in random orientations. Although in apoArgRC crystals the distance between the Cα atoms of Arg110 and Asp128 would permit their terminal functional groups to form a hydrogen-bonded salt bridge, no such salt bridges are detected; instead both functional groups make no intramolecular interactions but are surrounded by solvent density. However, no steric clash can be detected that would prevent a dihedral angle change enabling Arg110 to reach Asp128. These findings suggest that the high ion concentrations used in crystallization (50 mM NaHepes, 100 mM NaCl, 20 mM CaCl_2_; [14]) may interfere with salt bridge formation, trapping a high-energy state in which Arg110-Asp128 interactions are disrupted.

To clarify whether the Arg-Asp interaction is a cause or a consequence of apoArgRC rotation, an energy-minimized starting structure with Ala replacing all six Arg110 residues was created from the apoArgRC crystal structure, and equilibrated 20-ns simulations were analysed. The trajectories reveal no rotation of apoArgRC110Ala, implying that Arg110 promotes trimer rotation *via* interaction with Asp128. Re-introduction of Arg in place of Ala110 in any single subunit of apoArgRC110Ala does not support rotation but instead introduces local conformational changes by orienting toward residues Asp128-Asp129 across the pocket (not shown); incremental re-introduction of additional Arg residues into apoArgRC110Ala has not been investigated.

To examine the basis for the counterclockwise rotation reported in crystals from *M. tuberculosis*, 20-ns simulations were analysed for apoMtArgRC (PDB ID 2ZFZ) using parameters and preparation as for apoEcArgRC. Several simulations led to different, though equilibrated, states. Within some simulations two different conformations, rotated in opposite directions, are sampled (Figures S2 and S3); this observation indicates that the failure to observe counterclockwise rotation of apoEcArgRC is not due to any inherent limitation in the simulations. Clockwise rotation in the apoMtArgRC simulations is correlated with formation of Arg133-Asp146 salt bridges, the three-dimensional equivalent of EcArgRC Arg110-Asp128 (Figure 1D); counterclockwise rotation as observed in the MtArgRC crystal is triggered by Arg118, which salt-bridges from the opposite side of each pocket to Asp132 lying diagonally across. The EcArgRC residues equivalent to the Arg118-Asp132 pair of MtArgRC are His99 and Asp113, which presumably cannot promote rotation in the counterclockwise direction. As with EcArgRC, the terminal functional groups of each Arg-Asp pair are within contact distance in MtArgRC crystals, but no salt bridges are detected in either the rotated or non-rotated crystal conformation, again suggesting that the high-salt crystallization conditions (0.1 M Hepes, 0.1 M NaCl [10]) may interfere with salt-bridge formation.

The finding that MtArgRC rotates in both directions using two Arg-Asp pairs whereas EcArgRC rotates in one direction using a single pair suggests a general functional role of Arg residues in ArgR rotational dynamics. This hypothesis predicts that a mutated apoEcArgRC with His99 replaced by Arg, mimicking MtArgRC Arg118, should rotate in both directions. An energy-minimized His99Arg mutant structure was created from the apoEcArgRC crystal structure, and rotation in both directions was observed within one equilibrated 20-ns simulation, promoted by Arg99-Asp113 and Arg110-Asp128 salt bridges (not shown). The accessibility of both rotational directions during apoEcArgRCHis99Arg simulations further rules out inherent limitations of the simulation, and indicates that the directionality of rotation is governed solely by the directionality of the salt bridges. The hypothesis also predicts that rotation of apoBstArgR in the counterclockwise direction uses Arg97-Asp111, equivalent to MtArgRC Arg118-Asp132, but that rotation in the clockwise direction does not occur because BstArgR presents Val108 at the position of EcArgR Arg110 and has no other Arg residue nearby. Thus, EcArgR, MtArgR, and BstArgR are inferred to share a common global dynamic process in which rotation of trimers is driven by Arg-Asp ion pairing, even though none of these salt bridges is detected in the crystal structures.

### Conformational fluctuations of ArgRC

**Figure 2:**
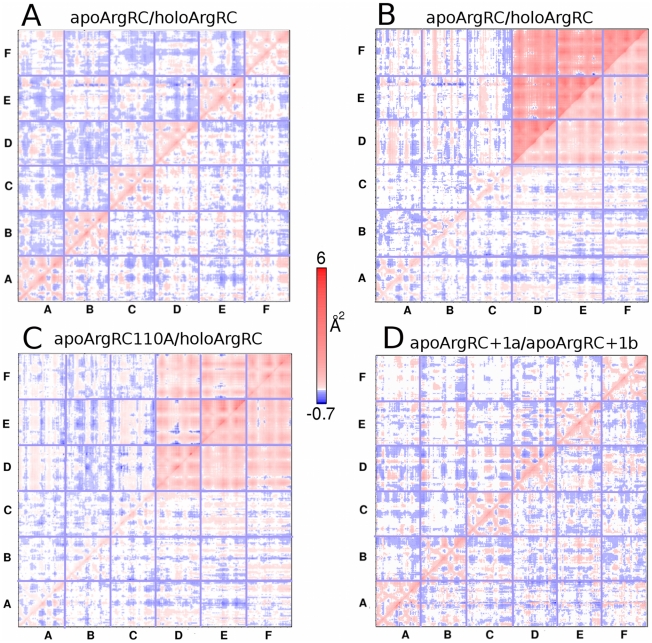
Correlated motions. The covariance matrix is defined as described in Materials and Methods. Vector products representing the maximum extent of correlated motion (Å^2^) for each C_α_ pair of each hexamer are plotted. The color scale indicates the degree of correlation: red, positively correlated; blue, negatively correlated; white, uncorrelated. Each panel presents two covariance matrices fused along the diagonal to eliminate redundancy and facilitate comparison of the two simulations indicated above the panel; the simulation presented in the upper left half is indicated first, followed by a slash (/) representing the diagonal, followed by the simulation in the lower right half. The reference state for calculating the extent of C_α_ motion as described in Materials and Methods is the hexamer for panels A and D and the trimer composed of ABC monomers for panels B and C.

Covariance analysis of Cα deviations during the equilibrated last 10 ns of each simulation reveals no clear pattern of correlated motions when the hexamer is the reference structure as defined in Materials and Methods (Figure 2A). Considering that trimers are involved in the early conformational shift, covariance analysis was referenced as described in Materials and Methods to the ABC trimer defined in Figure 1A to determine whether correlated motions of trimers occur during the simulation. Trimer referencing reveals dramatically correlated motions between apoArgRC trimers and slightly correlated motions between holoArgRC trimers (Figure 2B). Trimer referencing also unmasks an underlying pattern of traces reflecting the tertiary structure (detectable as nine small blocks within each red block), indicating that monomers remain folded during the simulations, as observed also in manual inspection of the trajectories. Principal-components analysis reveals that the dominant motion of apoArgRC trimers during the trajectory is rotational oscillation across the inter-trimer interface, accounting for the intense positive correlation. Thus, after the early rotational event in which apoArgRC rotates by 13° relative to the crystal structure, apoArgRC undergoes continuing rotational oscillation about the new mean structure. The relative motion between trimers of holoArgRC has no rotational component according to the results of principal-components analysis; the weaker correlation instead reflects small variations in inter-trimer distance along the three-fold axis. The covariance patterns of apoArgRC110Ala strongly resemble those of holoArgRC (Figure 2C), and those of apoMtArgRC resemble apoArgRC but reflect rotation in both directions (Figure S3).

**Figure 3:**
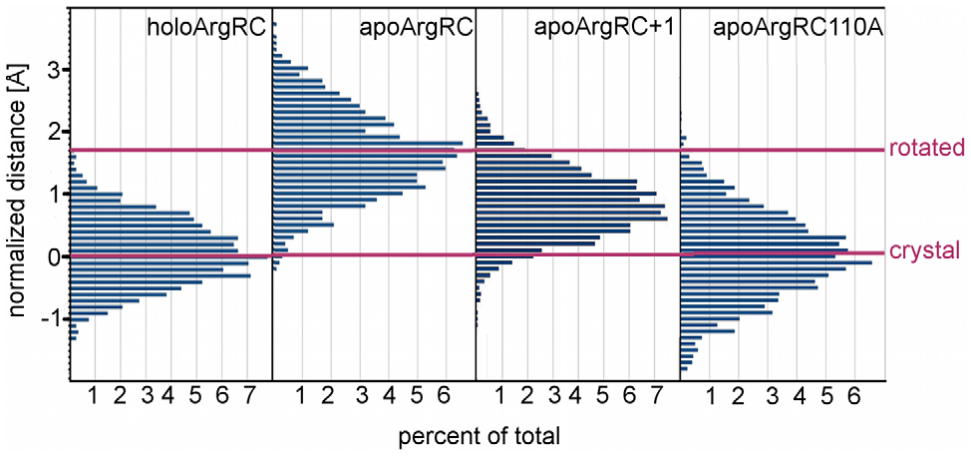
Species distribution. Distances between Gly103 and Asp128 C_α_ atoms are averaged over the six atom pairs in the hexamer every 50 ps during the final 10 ns of each indicated simulation. Distances are normalized by subtracting the average distance in holoArgRC crystals or, for apoArgRC110A, the distance in the energy-minimized starting structure. Distances are grouped by size in bins of 0.1 Å.

Residues lying along a line perpendicular to the axis of rotation were identified as metrics to quantify the conformational population distributions during each simulation. Gly103 of one monomer and Asp128 of the monomer diagonally across the binding pocket (Figure 1B) experience little local motion, but rotation of apoArgRC from the starting structure moves them apart by ∼1.6 Å. The six Gly103-Asp128 distances of each hexamer were measured during each trajectory (Figure S2) and normalized by subtracting the holoArgRC crystal distance (9.8 Å) to yield the distribution of distance deviations, Δδ, summarized in the frequency histograms of Figure 3. The histograms enable precise distinction between the initial rotation (the difference in the means of the distributions for apoArgRC and holoArgRC) and repetitive motions (the breadth of the distributions, reflecting rotational oscillation for apoArgRC and inter-trimer distance variation for holoArgRC). The holoArgRC ensemble with mean Δδ∼−0.1 Å samples mostly crystal-like distances, with a relatively narrow distribution. Similar results are found for apoArgRCArg110Ala, consistent with the results from covariance analysis (Figure 2C) and principal-components analysis that indicate the absence of rotational oscillation. Mean Δδ∼1.6 Å in the apoArgRC ensemble indicates that the rotated conformer is favored over the crystal-like conformer. During rotational oscillation apoArgRC samples a range of distances that at one extreme is equivalent to distances in the non-rotated starting state, but this extreme is sampled only rarely. ApoArgRC conformers interconvert freely with continuous change of energies, atom positions, and distances and a cycle time of ∼200–300 ps (not shown), suggesting the observed rotational oscillation represents natural hexamer motion driven by thermal flux with no energy barrier between conformers, i.e., motion about a local free energy minimum, with the rotated conformer lying at the bottom of the basin.

### Structural correlates of rotational oscillation

Inspection of the apoArgRC trajectory indicates that the dominant and rare conformers differ significantly in the average number of inter-trimer hydrogen bonds. In the dominant conformer all six Arg-Asp salt bridges are intact during the simulation and remain almost fully hydrogen bonded on average, due to an optimal approach distance of their terminal functional groups. In the rare crystal-like conformer one salt bridge per hexamer is broken on average, with Arg110 flipped outward from the binding pocket, and one other salt bridge lacks one or both of its hydrogen bonds due to a slightly increased distance between the functional groups, although electrostatic interaction is preserved. As a consequence of these differences in distances and hydrogen bond number, totaling ∼7–8 in the rare conformer and ∼10–11 in the dominant conformer, access of the L-arg ligand to its binding pockets also differs, with the rare conformer presenting one open binding site on average and the dominant conformer offering little access. Thus, thermal flux drives rotational oscillation of apoArgRC to transiently sample a conformation with one open L-arg binding site per hexamer. Although the ligand might occasionally sample this open site, the rotated conformation is not a thermodynamic state but rather represents only one extreme in a local free energy minimum; thus, because binding can occur only from a thermodynamic state, another, binding-competent, state must also exist.

The structure observed in apoArgRC crystals, with all Arg-Asp salt bridges broken but still within hydrogen-bonding distance, presumably represents a binding-competent state. Because it is never sampled again after the early rotational shift, even during long simulation times, this structure presumably lies at higher energy than the apoArgRC basin, but it must be accessible to apoArgRC even if it occurs rarely outside crystals, where lattice forces and/or crystallization conditions may favor it. However, apoArgRC crystals crack when L-arg is soaked in, apparently because the distance between Cα atoms of the salt-bridge pair is slightly larger in holoArgRC [14]. Thus an even higher-energy state of apoArgRC must also exist with Arg-Asp distances equal to those of holoArgRC; it is thus unclear if the binding-competent state resembles apo- or holoArgRC crystals. The finding that apoArgRC crystals crack upon addition of L-arg implies that crystal packing enforces the shorter Arg-Asp distance compatible with salt-bridge formation, suggesting that high-salt conditions alone interfere with salt-bridge formation.

ApoEcArgRC simulations prepared with additional sodium and chloride ions to mimic the crystallization conditions are consistent with this suggestion (not shown). At high concentrations sodium ions compete with Arg110 for interaction with Asp128, randomly disrupting salt bridges and replacing rotational oscillation with random motions. Ions coordinate with Asp129 as well, altering the orientation of the Asp128-129 pair and displacing Arg110 in random orientations. On average one subunit of the hexamer becomes more mobile than the other five. The latter observation suggests an interpretation of a puzzling crystallographic observation: apoArgR hexamers from **both** *B. stearothermophilus* [9] **and** *B. subtilis* [41] present good electron density for only five of the six identical subunits, a seemingly unusual coincidence. Enhanced mobility of one subunit might favor crystallization by offering an additional degree of freedom for the hexamer in the lattice. However, the structural features observed in the high-salt simulations might be unresolvable in fitting the experimental electron density due to the motions of the sidechains and solvent and the rotational degeneracy of ArgR.

### Simulations with L-arginine

Of 27 simulations initiated with artificial placement of ligands into apoArgR or their removal from holoArgR, L-arg maintained a crystal-like binding geometry after equilibration for two apoArgRC+1 (a and b), three holoArgRC-5 (a, b, and c), and three apoArgRC+2 simulations (a, b, and c); by this criterion the other 19 simulations were considered unsuccessful and were not analysed. None of the eight successful simulations experienced rotation or rotational oscillation in 20 ns, suggesting that binding of one L-arg per hexamer is sufficient to suppress rotational motions, consistent with its location spanning the inter-trimer interface. Covariance analysis, referenced therefore only to the hexamer to examine motions of individual monomers, reveals distinct patterns (Figure 2D). ApoArgRC+1a displays large regions within most monomers with uncorrelated motion, interspersed with regions of negatively correlated motions, suggesting that subunit motions become more random when L-arg binds, particularly for subunits BCF that in this simulation do not contact L-arg. The prominent tertiary traces despite very different overall extents of correlation within monomers indicate that no unfolding occurs and that internal motions are correlated with monomer motion. ApoArgRC+1b displays an approximately uniform and equal distribution of correlated and uncorrelated motions over all monomers, indicating local motions less correlated with monomer motion. All three holoArgRC-5 simulations are very similar to apoArgRC+1b. All singly-liganded simulations thus indicate that this state presents intense, largely random, motions of folded monomers within the hexamer.

Gly103-Asp128 distance histograms (Figure 3) reveal fluctuation in the two apoArgRC+1 simulations about a common mean structure similar to, but distinct from, that of holoArgRC. The range of the six individual inter-subunit distances is as narrow as the tightly clustered distances measured in holoArgRC (Figure S2), with a slightly narrower range for apoArgRC+1a and a slightly broader range for +1b and the three holoArgRC-5 simulations, due partly to larger local fluctuations. The narrow distribution of distances indicates that the intense random motions detected by covariance analysis are not reflected in motions at the trimer interface, and that hexamer symmetry is unexpectedly high. Symmetry in the +1arg simulations is further indicated by the distances from the center of mass of the hexamer to the center of mass of each monomer, which vary randomly during the entire equilibrated part of each simulation by less than the length of a covalent bond (mean distance ∼18±0.5 Å; Figure S2 and data not shown).

Addition of a second L-arg ligand, regardless of its position relative to the first, completes the conversion to fully holo-like mean distances, with similarly narrow range (not shown), indicating that the symmetry established in the +1arg state is maintained. Thus, binding of a single L-arg is necessary and sufficient to create an ensemble that is only slightly less holo-like than when all six sites are occupied, and in which symmetry is retained despite highly variable motions that reflect transfer of thermal flux to individual monomers. Importantly, the slightly greater Arg-Asp distances that are achieved only in the +2 state allow each of those residues to participate in electrostatic interactions with other surrounding residues, thus eliminating the directionality of motion.

In all five single-ligand simulations, the L-arg guanidino group forms a salt bridge to Asp128, replacing Arg110 that is displaced to make random motions; all other binding-site residues of the unliganded subunits maintain binding-competent conformations in all simulations. Four of the five single-ligand simulations present a common pattern, the +1a simulation being the exception. In those four, the unliganded binding pockets retain Arg110-Asp128 salt bridges, but their hydrogen bonding is frequently disrupted, and occasional opening of one further salt bridge is observed as well. The number of persistently populated inter-trimer hydrogen bonds (those present >50% of the time) is half or less of the total hydrogen bond number, indicating considerable flux in bonding partners. The ligand conformation is the same as in holoArgRC cocrystals, and it contributes one hydrogen bond. In contrast, in the +1a simulation the ligand conformation is fully extended, and it contributes two hydrogen bonds; however, the most striking feature of this simulation is that all inter-trimer hydrogen bonds are persistent, implying the existence of a cooperative hydrogen bonding network.

The ability of L-arg to compete successfully for interaction with Asp128 appears surprising considering that the effective local concentration of the residue sidechain is expected to be much higher. L-arg apparently wins the competition despite this disadvantage because unlike *residue* Arg110, *ligand* L-arg presents not only its guanidino group to Asp128 of one subunit, but its free α-amino and α-carboxylate substituents additionally form a complex mesh of interactions with eight more residues (Figure 1C): Gln106, Asp113, Thr124, and Ala126 in the same subunit that engages the guanidino group; Asp128, Asp129, and Thr130 in the subunit adjacent to the first in the same trimer; and Asp128 in a third subunit directly above the second in the other trimer; in the apoArgRC+1a simulation the ligand does not contact Gln106 but contacts Thr124 with higher frequency. Thus, each L-arg engages three subunits using common protein loop regions that cooperate to perform structurally distinct roles in the complex, creating a high-affinity site with few degrees of freedom for the ligand or the protein. The results indicate that L-arg ligands modulate global protein dynamics by competition with resident Arg residues for salt-bridge formation to Asp residues of the binding pockets.

### Energetic contributions and energy landscape

Per-ligand binding enthalpies and free energies, together with configurational entropy contributions to the total system free energy, were calculated as described in Materials and Methods from all simulations with zero, one, two, or six bound L-arg ligands (Table S1). Binding enthalpies per ligand are essentially the same for each single-ligand simulation as for each of the six ligands of holoArgRC, suggesting similar enthalpy increments for all six L-arg. Per-ligand binding affinities range widely for the singly-bound simulations, but all are significantly more favorable than the average value for holoArgRC, indicating that ligand affinity is substantially higher when only one L-arg is bound, consistent with ITC [16]. Entropies calculated from the covariance matrix are within error in all simulations; this finding is surprising considering that rotational oscillation is a major contributor to the entropy of apoArgRC but is absent in all states that include L-arg, indicating that these states must have other substantial sources of favorable entropy.

**Figure 4:**
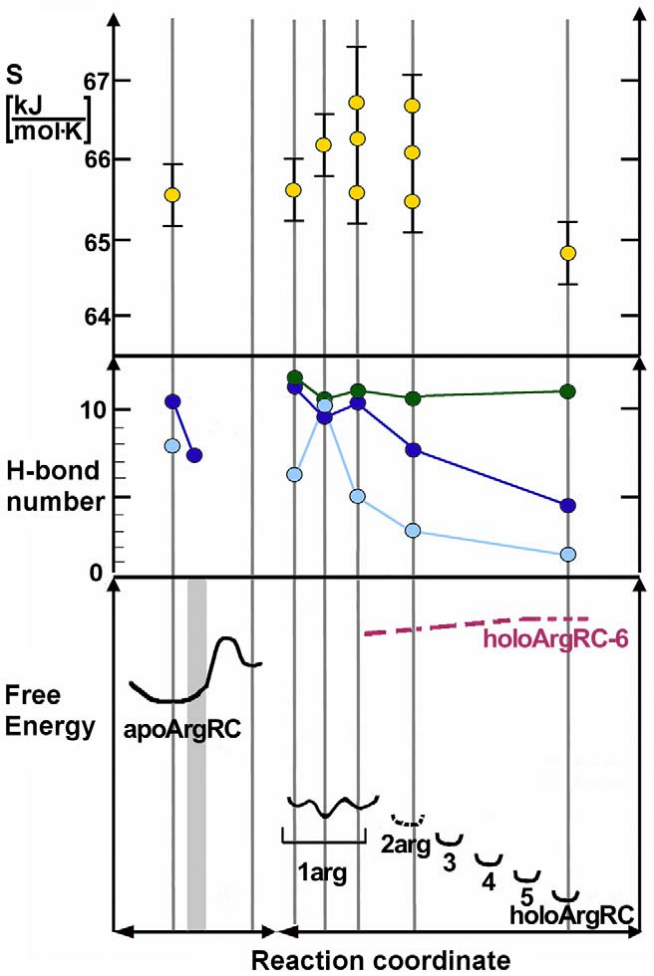
Reaction coordinate. Vertical lines mark coordinates for which values of conformational entropy, inter-trimer hydrogen bond number, and relative free energy are plotted based on inspection or calculation from the indicated simulation; second line from left marks the high-energy, binding-competent state based on inference as described in the text; grey zone in bottom panel indicates the rare conformer of apoArgRC. The three lines for the 1arg state represent (left to right) apoArgRC+1b, apoArgRC+1a, and the three holoArgRC-5 simulations. Top panel, conformational contributions to total system entropy (S, kJ/molK), calculated from each covariance matrix in which the reference state is the hexamer. Middle panel, average number of inter-trimer hydrogen bonds (filled circles) counted during each simulation (light blue, persistent bonds (present >50% of time); dark blue, total bond number not counting L-arg; green, total bond number counting L-arg; lines are only to guide the eye). Bottom panel, estimated relative free energy of the system. Estimates combine the values for entropy and hydrogen bond number given in the upper panels with the average per-ligand enthalpy and binding free energy values given in Table S1 (or where unavailable, the values calculated for holoArgRC). Estimated energy levels for ligands three, four, and five are obtained by linear interpolation between the levels for two and six bound L-arg ligands. The data do not uniquely constrain the energy level of the +2 state (dashed). HoloArgRC-6 simulations that did not equilibrate are inferred to approach the energy barrier from the right (red dashed line).

Relative free energy levels for each state were estimated by combining these energetic contributions with the numbers of inter-trimer hydrogen bonds, and the states were ordered along a reaction coordinate that accounts for all available information (Figure 4). The resulting free energy landscape is quite rough on its left half. On the apoArgRC conformational coordinate, freely oscillating apoArgRC and the crystal-like high-energy, binding-competent state are represented by a double minimum separated by a barrier, reflecting the fact that the starting state is not sampled again after the initial shift. Singly-liganded states are represented by a manifold with multiple minima, reflecting differences in the energetic contributions determined from the two +1arg and three -5arg simulations. Ligation states beyond +1 lie at progressively lower energy levels, reflecting the cumulative free energy lowering of successive ligand additions, with equal increments after +2arg, the energy level of which cannot be set with presently available information.

**Figure 5:**
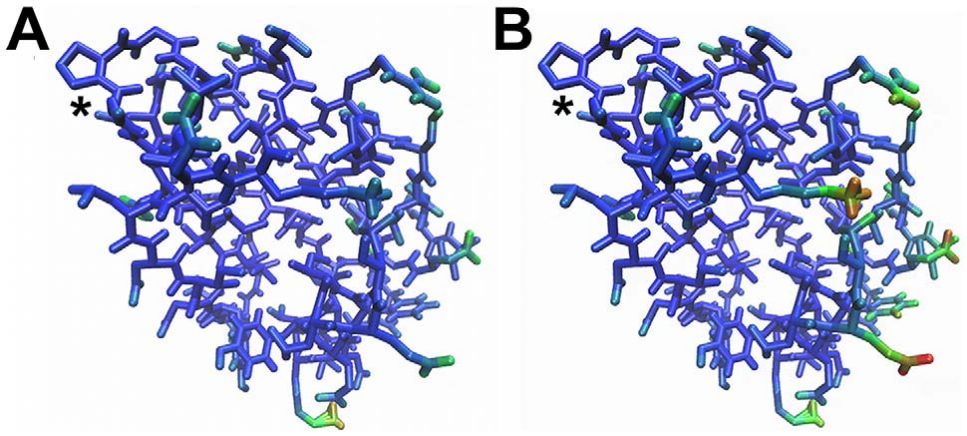
Motion gradient. View of one ArgRC subunit, with the L-arg binding site at the center of the hexamer at upper left (asterisk) and the outside of the hexamer at the right. The color gradient represents the extent of motion (blue, lowest to red, highest) defined by the root-mean-square displacement of each atom from its average position. Thickness of lines represents distance from the viewer (thickest in front). Left, apoArgRC; right, apoArgRC+1a.

The +1a simulation reveals the apparently self-contradictory result that entropy is undiminished even though all inter-trimer hydrogen bonds are persistent, i.e., the increased motions of individual subunits detected by covariance analysis are correlated with cooperative hydrogen bonding at the trimer interface. This result can be understood together with the other unexpected result for all +arg simulations: that hexamer symmetry is as high as in holoArgRC, as judged from the narrowly distributed Gly-Asp distances and essentially invariant center-of-mass distances. Such a seemingly paradoxical state, symmetric and with high entropy despite high hydrogen-bond occupancy, can be visualized as resulting from bonding constraints between the ligand and the subunits, as well as among subunits, that limit monomer motions close to the binding site but that transfer momentum to the peripheral parts of each subunit. This picture is confirmed by analysis of the root-mean-square displacement of each atom from its average position (Figure 5), showing that enhanced motion is confined to the surface, consistent with the patterns observed in covariance analysis. Although the differences between apoArgRC and +1arg states appear small in Figure 5, they are amplified in the hexamer by the contributions from all six subunits. Thus, by exploiting the dynamics of the assembly, all interactions between the ligand and the subunits, as well as among subunits, can be optimized simultaneously, generating maximum affinity in the +1 state through favorable contributions to both enthalpy and entropy.

### Integrated picture of ArgR allosteric mechanism

The following picture emerges of the structural, kinetic, and energetic events and their manifestation in ITC as L-arg binds to a population of rotationally oscillating apoArgRC hexamers. Free L-arg occasionally encounters a hexamer in a high-energy, non-rotated conformation (grey zone in Figure 4) with one open ligand-binding site and the remaining five salt bridges largely hydrogen-bonded. The highly charged L-arg ligand enters this site, where it may act similarly to high ion concentrations, promoting conversion to an even higher-energy, binding-competent state resembling crystalline apoArgRC, with Arg-Asp hydrogen bonds of the salt bridges mostly broken. Breaking of these hydrogen bonds constitutes an energy barrier between the apoArgRC basin and the binding-competent conformation, and is assigned to the slow ITC endotherm. The mechanism by which free L-arg promotes conversion to a binding-competent state, and the nature of this state, is under investigation to evaluate the interpretation suggested here.

Binding of one L-arg to the binding-competent conformation of apoArgRC releases the constraint on Arg-Asp hydrogen bonding. Arg110 residues in the empty binding sites engage in directional interactions with Asp128 residues, but bound L-arg acts as a brake on oscillation by steric interference. These opposing effects result in intense, random monomer motions propagated to the periphery of each subunit. At the center of the hexamer a cooperative hydrogen bond network is established among subunits and between subunits and ligand, optimizing affinity while maintaining symmetry. Thus the singly-liganded state is conceptually, but not structurally, asymmetric. Addition of a second L-arg forces a compromise in the optimized hydrogen bond network of the singly-liganded state, reducing binding energy by an unknown amount (dashed in Figure 4). The second ligand completes the conversion to a fully holo-like state with all salt bridges too distant to promote directional interactions, but with no further endothermic heat flow. The structural symmetry established in the singly-liganded state is preserved regardless of the placement of the second ligand relative to the first, because this symmetry is rooted in the global dynamics of the system rather than in its structural features.

The rate of L-arg dissociation from the singly-bound state is presumably slow relative to the time required for redistribution of the conformational ensemble; estimates from surface plasmon resonance [16] suggest an aggregate L-arg off-rate constant of ∼0.1 to 1.0 sec^−1^. Thus, when free L-arg enters an open binding site, as during the early stages of the ITC titration, part of the apoArgRC population slowly crosses the barrier to the binding-competent state, giving rise to a slow endotherm, and becomes trapped by L-arg binding; additions of further aliquots of L-arg repeat the cycle of barrier-crossing, endotherm evolution, and trapping until binding of the first equivalent of L-arg per hexamer is complete. A single equivalent of L-arg is thus necessary and sufficient to accomplish the shift of the dynamic quaternary ensemble. An allosteric mechanism originating in oscillatory dynamics of the C-terminal domain could account for the fact that both ArgR and ArgRC display identical 1+5 ligand-binding behaviors in ITC [16], suggesting that L-arg has the same global effect on the quaternary ensembles whether or not the DNA-binding domains are attached.

### Positive cooperativity, negative cooperativity, and symmetry

The features of the +1arg state appear to correspond to the prediction of the MWC model [17] that constraints arising from subunit assembly are relaxed upon ligand binding, leading to a high-affinity, monomer-like state, but with maintenance of symmetry during the conformational transition. Despite this consistency, the negative cooperativity of L-arg binding is incompatible with the MWC model, which predicts only positive cooperativity. Thus, like many other allosteric systems [42] including hemoglobin [43], ArgR appears to display features of both concerted and sequential models. However, the finding that symmetry can be preserved even during sequential filling of binding sites indicates the applicability of the symmetry principles of the MWC model to negative cooperativity, which its authors did not anticipate. At the time of the MWC model most cases of negative cooperativity were regarded as artifacts resulting from partial protein activity, as was later verified [44] for the controversial case of apparent negative cooperativity that had led to elaboration of the sequential allostery model [45]. Since that time, however, many carefully-documented examples of negative cooperativity including ArgR establish beyond doubt that both positive and negative cooperativity are common molecular strategies that serve complementary physiological purposes. Positive cooperativity enables a ligand to act as a switch by reducing the concentration of free ligand required to convert its target from the free to the bound state; negative cooperativity provides a buffer against changes in ligand concentration, requiring larger increases to convert the target from the free to the bound state.

The key enabling feature that makes ArgR cooperativity negative is the fact that conversion to the holo-like state proceeds in at least two ligand-binding steps, with affinity optimized in the first step and compromised in the second. The first ligand-binding step achieves maximal affinity by exploiting the dynamic nature of the protein to form a symmetric assembly in which all inter-subunit and ligand-subunit interactions are optimized simultaneously. The second ligand-binding step forces compromise among the interactions established in the first step, reducing ligand affinity and thereby conferring negative cooperativity. No obvious constraint demands that in other cases the two steps of optimization occur in the order observed for ArgR. Depending whether ligand affinity can be maximized in the first step as in ArgR or in subsequent steps, cooperativity is predicted to be either negative or positive, respectively.

The difference between positive and negative cooperativity presumably reflects the cohesiveness of the assembly at each ligand-binding step. Note that the free energy of a system is by definition a distributed property of the system, in which sources of cohesion arising from subunit-subunit or ligand-subunit interactions are not distinguished. Relatively weak subunit assemblies may be unable to optimize inter-subunit and ligand-subunit interactions in the first ligand-binding step if a single ligand makes an insufficient contribution to the cohesive free energy. In such cases subsequent ligands, rather than forcing a compromise as in the ArgR case, may take advantage of any partial relaxation promoted by the first ligand(s) to bind more strongly, yielding positive cooperativity. Thus, ligand-induced relaxation to a high-affinity, monomer-like state is limited by the cohesiveness of the assembly. As the MWC model points out, one of the advantageous properties associated with molecular symmetry [17] is that symmetric states allow equivalent interaction surfaces on all monomers, maximizing their cohesion. The ArgR example shows that symmetry can be maintained throughout the ligation process, even as optimized interactions are compromised.

Optimization in a single ligand-binding step may in fact be rare, as examples of positive cooperativity appear to vastly outnumber *bona fide* instances of negative cooperativity. The apparent preponderance of positive cooperativity implies that most assemblies lack sufficient cohesive free energy to permit optimization of inter-subunit and ligand-subunit interactions in a single ligand-binding step. This suggestion is consistent with the relatively weak subunit affinity that is common among protein multimers [46] and which is likely to be under selection pressure in order to preserve allosteric modulation. In many cooperative systems the free energies of subunit interaction are of similar magnitude as those of ligand interactions [47], indicating the two association processes are expected to exert mutual influence. Subunit affinities and their linkage to ligand binding has long been held to underlie the molecular mechanism of hemoglobin allostery [48], [49], although explicit correlation of its energetic and dynamic structural pictures has begun only recently [50]. Thus, the magnitude of inter-subunit affinity relative to ligand affinity is expected to predict whether a system exhibits positive or negative cooperativity. Subunit affinity is extremely high for apoArgR; for hexamer-trimer dissociation only an upper-limit value, K_d_≤2.5 nM, is available from analytical ultracentrifugation data [2].

### Speculations on the evolution of allostery

An archetypal ArgR presumably acquired residues that enabled a non-covalent ligand to substitute for its covalent counterpart, permitting feedback control by the regulon end-product. The work required to oscillate across the apoEcArgRC trimer interface is apparently small relative to the strength of Arg110-Asp128 interactions. However, the extremely high conservation of Arg-Asp pairs in the binding sites of ArgR orthologs contrasts with substantial differences in residues at their trimer interfaces identified by multiple sequence alignment (not shown). This finding may account for the apparently divergent behaviors of ArgR orthologs with respect to the effects of L-arg binding. If competition between resident sidechains and L-arg ligands is general among ArgRs, the balance between oscillation work and strength of Arg-Asp pairs may be tuned differently in orthologs that occupy varied ecological niches.

Covalent/non-covalent substitutions may be a general path to a useful response, and a particularly effective evolutionary driver of allostery among amino acid-binding proteins, as suggested by a second feedback regulator that uses the principle in the opposite sense. *E. coli* tryptophan repressor, TrpR, presents Gly85 instead of a hydrophobic residue, often Trp, found at the DNA-binding interface of other helix-turn-helix proteins [51]. The small size of Gly85 helps to accommodate the co-effector L-trp [52]. An archetypal TrpR presumably lost the aromatic residue at this position, creating the L-trp binding site and perhaps playing a role in evolution of domain-swapped TrpR _haract [53] that present a network of contacts from both subunits to each L-trp ligand to bring DNA binding under control of the regulon end product. Thus, amino acid binding by ArgR and TrpR recalls, and extends to allostery, the ambiguity of the boundary between covalent and non-covalent processes in proteins that is inherent in protein folding, which relies on cooperation between the covalent primary structure and the weak non-covalent interactions that couple the secondary structure to the tertiary structure [53]. Given its simplicity, amino acid binding may have been an early innovation in the evolution of allostery; similar covalent/non-covalent substitutions are known among nucleotide-binding RNAs [54], [55], suggesting that ligand/residue substitution, which requires the existence of only polymers and their constituent monomers, could predate the evolution of protein subunit assemblies that exploited the innovation for homotropic cooperativity. Indeed, the subtle modulation of protein activity by monomer binding, compared with the all-or-none effects on RNAs [54]–[56], may have played a role in the ascendancy of proteins in the RNA world.

The vast majority of allosteric ligands do not correspond to the monomeric constituents of their targets, and some proteins can respond even to non-biological ligands, representing an extreme example of gratuity as originally defined by Monod [3], [57], [58]: the concept that effectors need not resemble substrates, as exemplified by, e.g., the inducer of the *lac* operon, isopropylthiogalactoside. A particularly dramatic example is bacteriophage T4 lysozyme, where single-residue substitutions produce binding sites for benzene [59]. Presumably, selection pressure for allosterically responsive targets can be created by any ligand exploiting any evolutionarily intermediate binding site on any macromolecule. Thus all binding species should probably be considered as potential allosteric effectors, reflecting the enormous capacity for allosteric response that is likely to be inherent in nearly all intermolecular interactions, just as allostery is understood to be a universal property of dynamic proteins [17].

## Supporting Information

Figure S1 Time course of simulations. Every 50 ps during the trajectory, the root-mean-square deviation (RMSD, Å) of Cα positions is derived by overlaying each simulation structure with its corresponding initial structure by Cα superposition. Each color represents the individual simulation indicated. Note the compressed time scale after 20 ns.
(0.55 MB PDF)

Figure S2 Gly103-Asp128 distances. Every 50 ps during the final 10 ns of each indicated simulation, distances between Gly103 and Asp128 residues are measured for each of the six pairs in the hexamer. Monomer colors (A, black; B, red; C, green; D, blue; E, cyan; F, magenta) correspond to those of Figure 1A except that subunit A is black for better visualization, and the one outlier in apoArgRC due to local conformational change is grey.
(1.05 MB PDF)

Figure S3 Correlated motions of *M. tuberculosis* apoArgRC. The covariance color-scale values are doubled relative to Figure 2 of the main text because *M. tuberculosis* apoArgRC rotates in both directions. The reference state is the hexamer; all other details are as described in the legend to main text Figure 2.
(0.87 MB PDF)

Table S1 Energetic contributions. Each row corresponds to a separate simulation.
(0.04 MB PDF)
