# Supplementary figures and images for: Symmetric Allosteric Mechanism of Hexameric Escherichia coli Arginine Repressor Exploits Competition between L-Arginine Ligands and Resident Arginine Residues
Source: PLoS Comput Biol. 2010 Jun 3;6(6):e1000801. doi: 10.1371/journal.pcbi.1000801 (PMC2880562; doi:10.1371/journal.pcbi.1000801)

**Figure S1. Time course of simulations.**

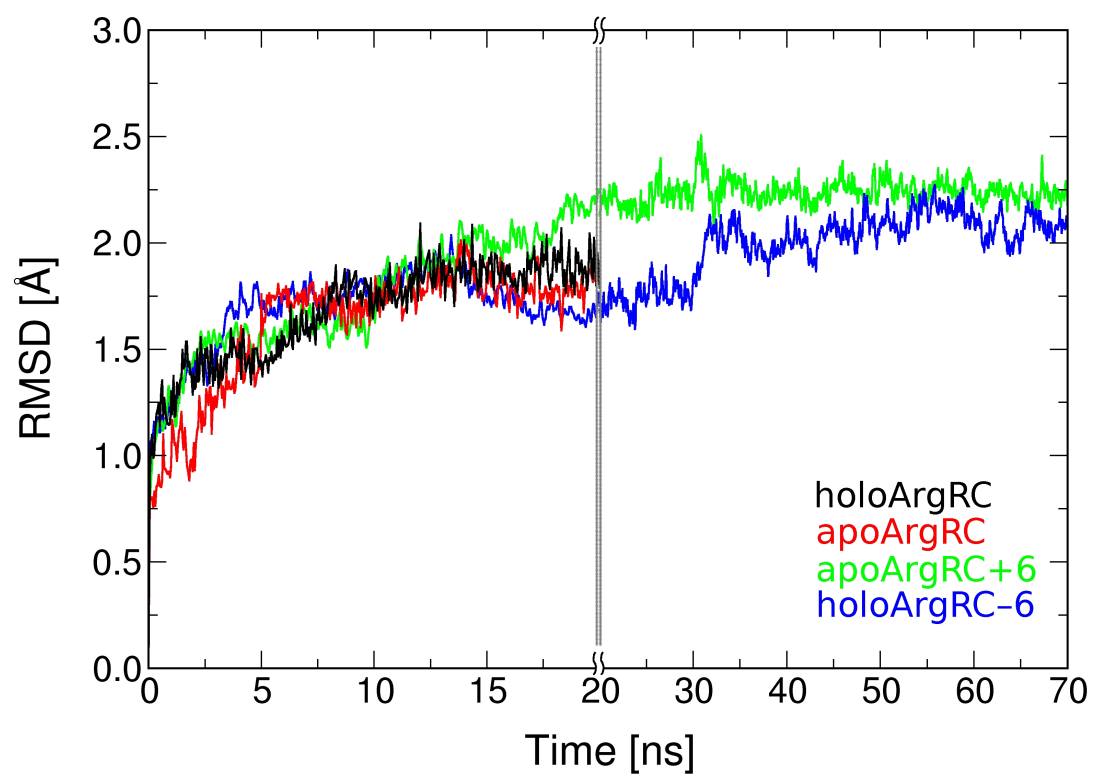

Supplement: Figure S1 — Time course of simulations. Every 50 ps during the trajectory, the root-mean-square deviation (RMSD, Å) of Cα positions is derived by overlaying each simulation structure with its corresponding initial structure by Cα superposition. Each color represents the individual simulation indicated. Note the compressed time scale after 20 ns. (0.55 MB PDF) [file pcbi.1000801.s001.pdf]

**Figure S2. Gly103-Asp128 distances.**

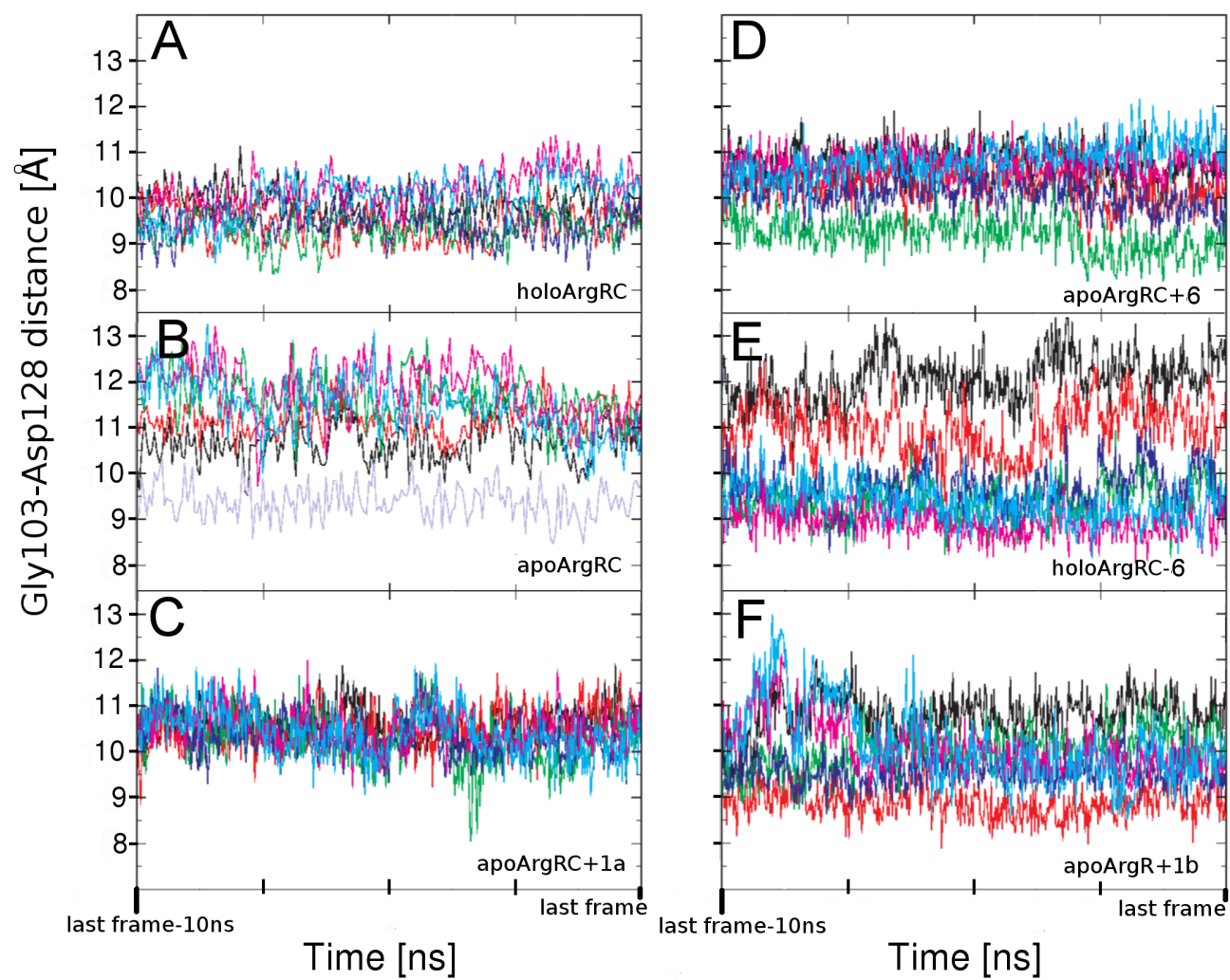

Supplement: Figure S2 — Gly103-Asp128 distances. Every 50 ps during the final 10 ns of each indicated simulation, distances between Gly103 and Asp128 residues are measured for each of the six pairs in the hexamer. Monomer colors (A, black; B, red; C, green; D, blue; E, cyan; F, magenta) correspond to those of Figure 1A except that subunit A is black for better visualization, and the one outlier in apoArgRC due to local conformational change is grey. (1.05 MB PDF) [file pcbi.1000801.s002.pdf]

**Figure S3. Correlated motions of *M. tuberculosis* apoArgRC.**

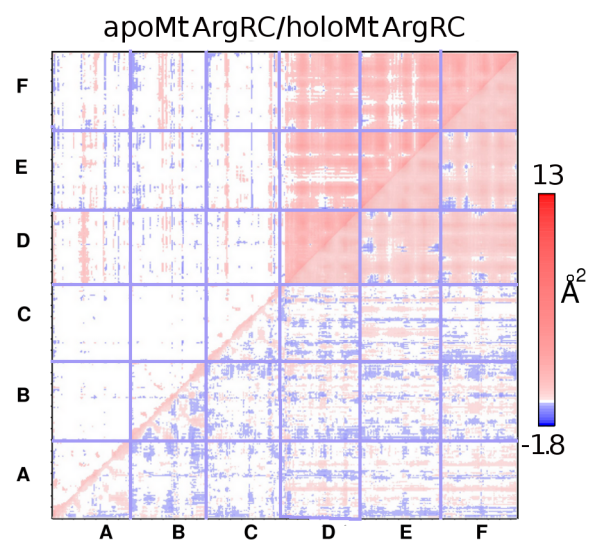

Supplement: Figure S3 — Correlated motions of M. tuberculosis apoArgRC. The covariance color-scale values are doubled relative to Figure 2 of the main text because M. tuberculosis apoArgRC rotates in both directions. The reference state is the hexamer; all other details are as described in the legend to main text Figure 2. (0.87 MB PDF) [file pcbi.1000801.s003.pdf]
