# Supplementary material for: Symmetric Allosteric Mechanism of Hexameric Escherichia coli Arginine Repressor Exploits Competition between L-Arginine Ligands and Resident Arginine Residues
Source: PLoS Comput Biol. 2010 Jun 3;6(6):e1000801. doi: 10.1371/journal.pcbi.1000801 (PMC2880562; doi:10.1371/journal.pcbi.1000801)
Supplement: Table S1 — Energetic contributions. Each row corresponds to a separate simulation. (0.04 MB PDF) [file pcbi.1000801.s004.pdf]

Table S1. Energetic contributions.

Simulation	System conformational entropy, kJ/molK		Binding enthalpy per ligand, kJ/mol	Binding free energy per ligand, kJ/mol
holoArgRC	64.8	± 0.4	-733 ± 198	-241 ± 38
apoArgRC	65.6	± 0.3		
holoArgRC – 5a L-arg	66.7	± 0.7	-873	-415
holoArgRC – 5b L-arg	65.6	± 0.3	-696	-313
holoArgRC – 5c L-arg	66.3	± 0.2	-816	-377
apoArgRC + 1a L-arg	66.2	± 0.3	-671	-318
apoArgRC + 1b L-arg	65.6	± 0.3	-818	-379
apoArgRC + 2a L-arg	66.1	± 0.4	not determined	-237
apoArgRC + 2b L-arg	66.7	± 0.3	not determined	-264
apoArgRC + 2c L-arg	65.4	± 0.2	not determined	-295
